# 2-Methyl-3-(10*H*-pheno­thia­zin-10-yl)buta-1,3-diene-1,1,4,4-tetra­carbo­nitrile

**DOI:** 10.1107/S1600536813008799

**Published:** 2013-04-05

**Authors:** Tsunehisa Okuno, Hirokazu Iwahashi

**Affiliations:** a Department of Material Science and Chemistry, Wakayama University, Sakaedani, Wakayama 640-8510, Japan

## Abstract

In the title compound, C_21_H_11_N_5_S, the pheno­thia­zine unit has a butterfly structure, and the central six-membered ring adopts a boat conformation. The dihedral angle between the benzene rings is 127.64 (6)°, which is smaller than those reported for similar compounds because of the steric repulsion between the pheno­thia­zine and its tetra­cyano-1,3-butadiene substituent. The di­cyano­vinyl groups are almost orthogonal to one another, making a dihedral angle of 80.58 (6)°. In the crystal, the mol­ecules are aligned along the *b* axis. Four kinds of weak C—H⋯N inter­actions are recognized, one of which connects the mol­ecules into a one-dimensional array and the remaining three link these arrays.

## Related literature

For applications of tetra­cyano-1,3-butadienes in photonics and non-linear optics, see: Faupel *et al.* (2007 ▶). For the preparation and structure of 10-(prop-1-yn-1-yl)-10*H*-pheno­thia­zine, see: Zaugg *et al.* (1958 ▶); Umezono & Okuno (2012 ▶). For the structures of other related *N*-substituted pheno­thia­zines, see: Chu & Van der Helm (1974 ▶, 1975 ▶); Tokunaga & Okuno (2012 ▶).
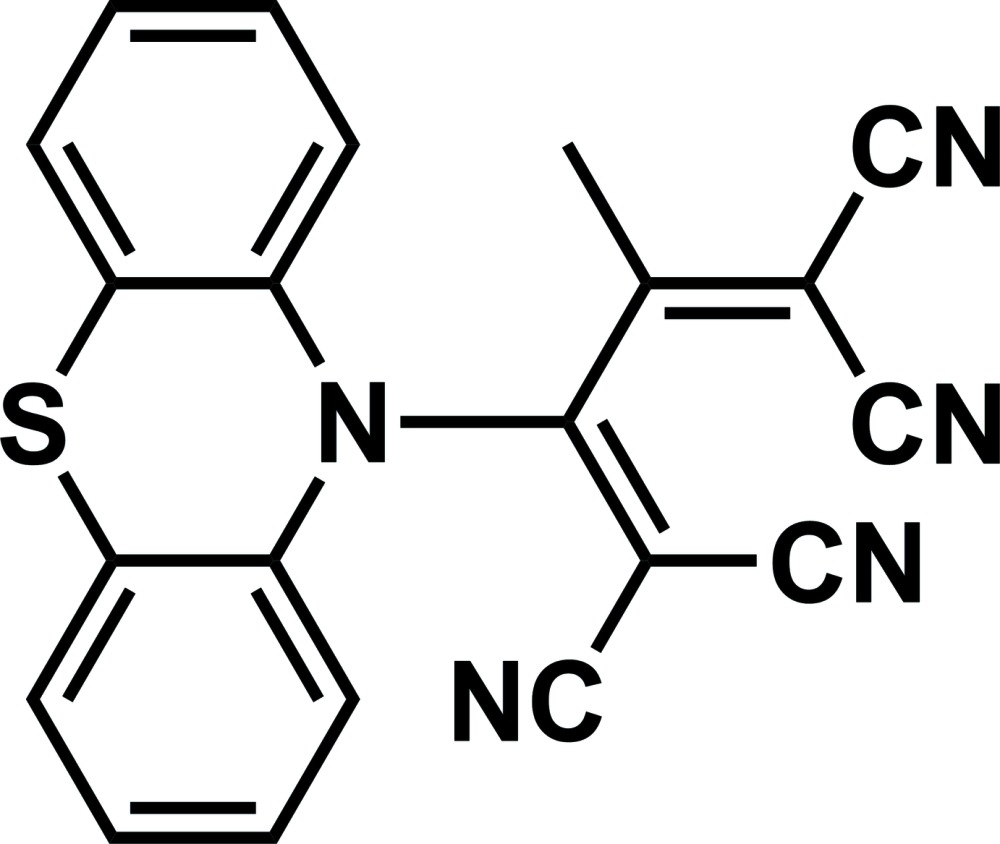


## Experimental

### 

#### Crystal data

C_21_H_11_N_5_S
*M* *_r_* = 365.41
Monoclinic, 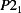

*a* = 10.217 (3) Å
*b* = 7.848 (3) Å
*c* = 11.369 (3) Å
β = 97.316 (4)°
*V* = 904.2 (5) Å^3^
*Z* = 2
Mo *K*α radiation
μ = 0.19 mm^−1^
*T* = 93 K
0.10 × 0.10 × 0.05 mm

#### Data collection

Rigaku Saturn724+ diffractometer
7524 measured reflections
3753 independent reflections
3494 reflections with *F* ^2^ > 2σ(*F* ^2^)
*R*_int_ = 0.022

#### Refinement

*R*[*F* ^2^ > 2σ(*F* ^2^)] = 0.032
*wR*(*F* ^2^) = 0.078
*S* = 1.04
3753 reflections
244 parameters
1 restraint
H-atom parameters constrained
Δρ_max_ = 0.21 e Å^−3^
Δρ_min_ = −0.25 e Å^−3^
Absolute structure: Flack (1983 ▶), 1526 Friedel pairs
Flack parameter: 0.01 (6)

### 

Data collection: *CrystalClear* (Rigaku, 2008 ▶); cell refinement: *CrystalClear*; data reduction: *CrystalClear*; program(s) used to solve structure: *SIR92* (Altomare *et al.*, 1994 ▶); program(s) used to refine structure: *SHELXL97* (Sheldrick, 2008 ▶); molecular graphics: *ORTEP-3 for Windows* (Farrugia, 2012 ▶); software used to prepare material for publication: *CrystalStructure* (Rigaku, 2010 ▶).

## Supplementary Material

Crystal structure: contains datablock(s) global, I. DOI: 10.1107/S1600536813008799/sj5308sup1.cif

Structure factors: contains datablock(s) I. DOI: 10.1107/S1600536813008799/sj5308Isup2.hkl

Supplementary material file. DOI: 10.1107/S1600536813008799/sj5308Isup3.cml

Additional supplementary materials:  crystallographic information; 3D view; checkCIF report

## Figures and Tables

**Table 1:** Hydrogen-bond geometry (Å, °)

*D*—H⋯*A*	*D*—H	H⋯*A*	*D*⋯*A*	*D*—H⋯*A*
C15—H15*B*⋯N4^i^	0.98	2.65	3.186 (3)	114
C2—H2⋯N5^ii^	0.95	2.59	3.352 (3)	137
C10—H10⋯N2^iii^	0.95	2.70	3.413 (3)	133
C8—H8⋯N2^iv^	0.95	2.62	3.480 (3)	151

Symmetry codes: (i) 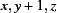; (ii) 
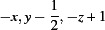
; (iii) 
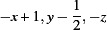
; (iv) 
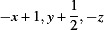
.

**Fig. 1.**
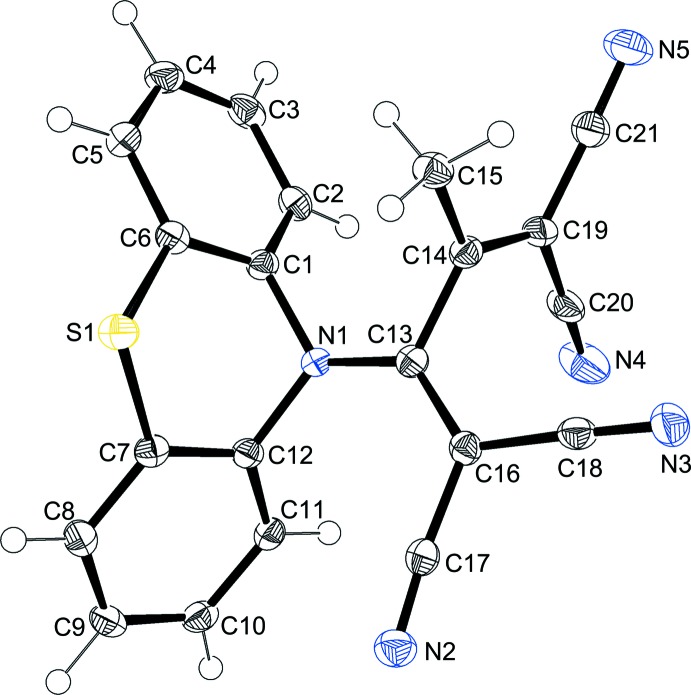
The asymmetric unit of the title compound with atom-numbering scheme. Displacement ellipsoids are drawn at the 50% probability level and H atoms are shown as small spheres.

**Fig. 2.**
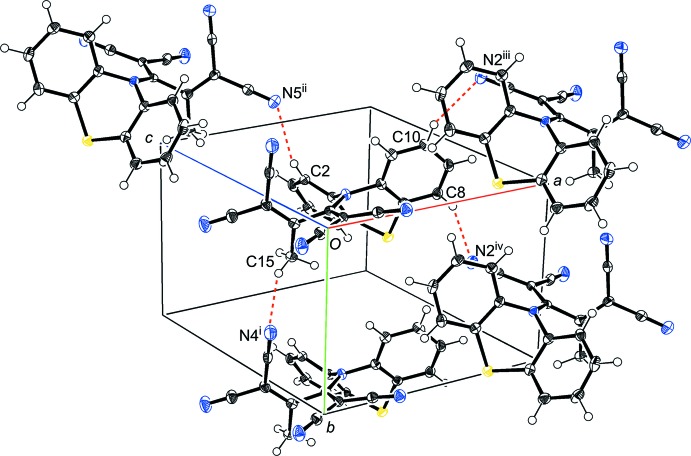
A view of the intermolecular interactions of the title compound. [Symmetry codes: (i) x, y + 1, z (ii) -x, y - 1/2, -z + 1 (iii) -x + 1, y - 1/2, -z (iv) -x + 1, y + 1/2, -z.]

## supplementary crystallographic information

### Comment

Tetracyano-1,3-butadienes, which are prepared by TCNE addition to acetylene compounds carrying an electron donating part, have been attracted interest from the viewpoint of applications in photonics and nonlinear optics (Faupel *et al.*, 2007). The title compound, C_21_H_11_N_5_S_1_, is a TCNE adduct of 10-(prop-1-yn-1-yl)-10*H*-phenothiazine which is known as the first ynamine compound (Zaugg *et al.*, 1958).

The phenothiazine moiety has a butterfly structure, and the central six-membered ring adopts a boat conformation (Fig. 1). The dihedral angle between the C1—C6 and C7—C12 planes is 127.64 (6)°. This is smaller than angles reported for related phenothiazine systems (Chu & Helm, 1974, 1975; Umezono & Okuno, 2012; Tokunaga & Okuno, 2012), and is presumably because of the steric repulsion between the phenothiazine ring system and its tetracyano-1,3-butadiene substituent. The structure around the N1 is pyrimidal and the N1 atom lies 0.1440 (14) Å out of the C1/C12/C13 plane. This plane is inclined at 10.84 (4)° to the C13/C16—C18/N2/N3 dicyanovinyl plane (r.m.s. deviation = 0.0095 Å), indicating good π-conjugation. This latter plane is approximately orthogonal to the other C14/C19—C21/N4/N5 plane (r.m.s. deviation = 0.0170 Å), with a dihedral angle of 80.58 (6)° between them.

The molecules align along the *b* axis. Four kinds of weak C—H···N interactions are recognized in each molecule, one of which connects the molecules within the one-dimensional array and the ramaining three link these arrays (Fig. 2, Table 1).

### Experimental

A solution of 10-(prop-1-yn-1-yl)-10*H*-phenothiazine (0.50 g, 2.1 mmol) (Zaugg *et al.*, 1958) in dichloromethane (25 ml) was added to a solution of ethene-1,1,2,2-tetracarbonitrile (0.27 g, 2.1 mmol) in dichloromethane (150 ml). The solution was stirred for a day and was concentrated by evaporation. The residue was purified by gel permeation chromatography (GPC) to give the title compound as pale blue powder (0.48 g, 62%). Single crystals with sufficient quality for X-ray crystallographic analysis were prepared by recrystallization from an acetonitrile solution.

### Refinement

The C-bound H atoms were placed at ideal positions and were refined as riding on their parent C atoms. *U*_iso_(H) values of the H atoms were set at 1.2*U*_eq_(parent atom).

### Crystal data

**Table:**

C_21_H_11_N_5_S	*F*(000) = 376.00
*M**_r_* = 365.41	*D*_x_ = 1.342 Mg m^−^^3^
Monoclinic, *P*2_1_	Mo *K*α radiation, λ = 0.71075 Å
Hall symbol: P 2yb	Cell parameters from 3683 reflections
*a* = 10.217 (3) Å	θ = 1.8–30.7°
*b* = 7.848 (3) Å	µ = 0.19 mm^−^^1^
*c* = 11.369 (3) Å	*T* = 93 K
β = 97.316 (4)°	Block, pale blue
*V* = 904.2 (5) Å^3^	0.10 × 0.10 × 0.05 mm
*Z* = 2	

### Data collection

**Table:**

Rigaku Saturn724+ diffractometer	*R*_int_ = 0.022
Detector resolution: 28.445 pixels mm^-1^	θ_max_ = 27.5°
ω scans	*h* = −12→13
7524 measured reflections	*k* = −9→10
3753 independent reflections	*l* = −14→14
3494 reflections with *F*^2^ > 2σ(*F*^2^)	

### Refinement

**Table:**

Refinement on *F*^2^	Hydrogen site location: inferred from neighbouring sites
*R*[*F*^2^ > 2σ(*F*^2^)] = 0.032	H-atom parameters constrained
*wR*(*F*^2^) = 0.078	*w* = 1/[σ^2^(*F*_o_^2^) + (0.045*P*)^2^ + 0.1327*P*] where *P* = (*F*_o_^2^ + 2*F*_c_^2^)/3
*S* = 1.04	(Δ/σ)_max_ < 0.001
3753 reflections	Δρ_max_ = 0.21 e Å^−^^3^
244 parameters	Δρ_min_ = −0.25 e Å^−^^3^
1 restraint	Absolute structure: Flack (1983), 1526 Friedel pairs
Primary atom site location: structure-invariant direct methods	Flack parameter: 0.01 (6)
Secondary atom site location: difference Fourier map	

### Special details

**Table:**

Refinement. Refinement was performed using all reflections. The weighted *R*-factor (*wR*) and goodness of fit (*S*) are based on *F*^2^. *R*-factor (gt) are based on *F*. The threshold expression of *F*^2^ > 2.0 σ(*F*^2^) is used only for calculating *R*-factor (gt).

### Fractional atomic coordinates and isotropic or equivalent isotropic displacement parameters (Å^2^)

**Table:**

	*x*	*y*	*z*	*U*_iso_*/*U*_eq_	
S1	0.46875 (4)	0.28150 (5)	0.24265 (4)	0.02108 (10)	
N1	0.27521 (13)	0.00720 (18)	0.24818 (12)	0.0165 (3)	
N2	0.25743 (15)	−0.0784 (3)	−0.09700 (14)	0.0250 (4)	
N3	−0.12618 (16)	0.0506 (3)	−0.02155 (14)	0.0300 (4)	
N4	−0.03764 (19)	−0.3121 (3)	0.2637 (2)	0.0404 (5)	
N5	−0.20384 (18)	0.1361 (3)	0.42695 (17)	0.0368 (5)	
C1	0.30948 (16)	0.0842 (3)	0.36326 (14)	0.0173 (4)	
C2	0.26062 (16)	0.0219 (3)	0.46368 (14)	0.0192 (4)	
C3	0.29033 (17)	0.1081 (3)	0.57034 (15)	0.0239 (4)	
C4	0.36780 (17)	0.2539 (3)	0.57534 (15)	0.0253 (4)	
C5	0.42255 (17)	0.3100 (3)	0.47617 (16)	0.0235 (4)	
C6	0.39519 (16)	0.2229 (3)	0.36953 (14)	0.0181 (4)	
C7	0.48704 (16)	0.0732 (3)	0.18623 (13)	0.0167 (4)	
C8	0.59958 (17)	0.0265 (3)	0.13638 (14)	0.0199 (4)	
C9	0.61205 (17)	−0.1403 (3)	0.09858 (15)	0.0209 (4)	
C10	0.51615 (17)	−0.2620 (3)	0.11218 (15)	0.0210 (4)	
C11	0.40235 (15)	−0.2157 (3)	0.16050 (13)	0.0190 (3)	
C12	0.38819 (16)	−0.0473 (3)	0.19417 (14)	0.0165 (4)	
C13	0.15512 (16)	0.0349 (2)	0.18619 (14)	0.0162 (4)	
C14	0.05534 (16)	0.1133 (3)	0.25659 (14)	0.0180 (4)	
C15	0.05982 (17)	0.3019 (3)	0.27492 (15)	0.0218 (4)	
C16	0.11722 (16)	0.0065 (3)	0.06727 (15)	0.0169 (4)	
C17	0.19996 (17)	−0.0424 (3)	−0.01964 (15)	0.0190 (4)	
C18	−0.01790 (17)	0.0327 (3)	0.01951 (14)	0.0208 (4)	
C19	−0.03508 (17)	0.0124 (3)	0.29768 (15)	0.0202 (4)	
C20	−0.03738 (18)	−0.1681 (3)	0.27904 (18)	0.0253 (4)	
C21	−0.13076 (18)	0.0814 (3)	0.36816 (17)	0.0260 (4)	
H2	0.2078	−0.0780	0.4593	0.0231*	
H3	0.2577	0.0674	0.6398	0.0287*	
H4	0.3837	0.3162	0.6474	0.0303*	
H5	0.4783	0.4073	0.4814	0.0282*	
H8	0.6668	0.1077	0.1284	0.0239*	
H9	0.6875	−0.1719	0.0627	0.0250*	
H10	0.5281	−0.3765	0.0886	0.0252*	
H11	0.3361	−0.2976	0.1702	0.0228*	
H15A	0.0728	0.3268	0.3601	0.0261*	
H15B	−0.0234	0.3525	0.2389	0.0261*	
H15C	0.1330	0.3500	0.2378	0.0261*	

### Atomic displacement parameters (Å^2^)

**Table:**

	*U*^11^	*U*^22^	*U*^33^	*U*^12^	*U*^13^	*U*^23^
S1	0.0262 (2)	0.0173 (2)	0.0209 (2)	−0.00254 (18)	0.00752 (15)	0.00042 (16)
N1	0.0147 (7)	0.0192 (8)	0.0154 (7)	−0.0000 (6)	0.0014 (5)	−0.0034 (6)
N2	0.0252 (8)	0.0294 (9)	0.0208 (8)	0.0023 (7)	0.0040 (6)	−0.0002 (7)
N3	0.0214 (8)	0.0444 (11)	0.0240 (8)	0.0023 (8)	0.0017 (7)	−0.0006 (7)
N4	0.0411 (11)	0.0224 (10)	0.0632 (14)	−0.0058 (8)	0.0282 (10)	−0.0079 (9)
N5	0.0355 (10)	0.0319 (11)	0.0470 (11)	−0.0014 (8)	0.0204 (9)	−0.0128 (8)
C1	0.0180 (8)	0.0184 (9)	0.0151 (8)	0.0022 (7)	0.0008 (6)	−0.0008 (7)
C2	0.0175 (8)	0.0192 (9)	0.0215 (8)	0.0015 (7)	0.0040 (7)	0.0028 (7)
C3	0.0217 (9)	0.0338 (11)	0.0168 (8)	0.0078 (8)	0.0047 (7)	0.0035 (7)
C4	0.0232 (9)	0.0339 (12)	0.0180 (8)	0.0067 (8)	−0.0003 (7)	−0.0069 (8)
C5	0.0203 (8)	0.0246 (11)	0.0251 (9)	−0.0011 (8)	0.0009 (7)	−0.0074 (7)
C6	0.0180 (8)	0.0194 (8)	0.0171 (8)	0.0018 (7)	0.0030 (7)	0.0009 (6)
C7	0.0187 (8)	0.0174 (8)	0.0136 (7)	0.0018 (7)	0.0002 (6)	0.0012 (6)
C8	0.0175 (8)	0.0266 (9)	0.0154 (8)	0.0004 (8)	0.0018 (6)	0.0037 (7)
C9	0.0183 (8)	0.0294 (10)	0.0152 (8)	0.0064 (8)	0.0034 (6)	0.0018 (7)
C10	0.0221 (9)	0.0224 (10)	0.0172 (8)	0.0048 (7)	−0.0020 (7)	−0.0039 (6)
C11	0.0177 (8)	0.0210 (8)	0.0175 (7)	0.0000 (8)	−0.0009 (6)	−0.0019 (7)
C12	0.0156 (8)	0.0210 (8)	0.0126 (7)	0.0030 (7)	0.0002 (6)	−0.0011 (6)
C13	0.0180 (8)	0.0109 (8)	0.0199 (8)	−0.0016 (7)	0.0033 (6)	−0.0001 (6)
C14	0.0188 (9)	0.0173 (9)	0.0173 (8)	0.0015 (7)	−0.0001 (6)	−0.0016 (6)
C15	0.0243 (9)	0.0173 (10)	0.0244 (8)	0.0010 (8)	0.0057 (7)	−0.0003 (7)
C16	0.0159 (8)	0.0155 (8)	0.0195 (8)	−0.0017 (7)	0.0033 (6)	0.0002 (7)
C17	0.0184 (8)	0.0193 (9)	0.0186 (8)	−0.0000 (7)	0.0003 (7)	0.0007 (6)
C18	0.0230 (10)	0.0238 (9)	0.0159 (8)	0.0027 (8)	0.0037 (7)	−0.0016 (7)
C19	0.0197 (8)	0.0179 (9)	0.0234 (8)	0.0010 (7)	0.0045 (7)	−0.0050 (7)
C20	0.0208 (9)	0.0246 (10)	0.0331 (10)	−0.0042 (7)	0.0139 (8)	−0.0049 (8)
C21	0.0234 (10)	0.0231 (10)	0.0329 (10)	−0.0047 (8)	0.0090 (8)	−0.0066 (8)

### Geometric parameters (Å, º)

**Table:**

S1—C6	1.7701 (18)	C11—C12	1.389 (3)
S1—C7	1.7745 (19)	C13—C14	1.505 (3)
N1—C1	1.443 (2)	C13—C16	1.376 (3)
N1—C12	1.440 (3)	C14—C15	1.495 (3)
N1—C13	1.352 (2)	C14—C19	1.345 (3)
N2—C17	1.153 (3)	C16—C17	1.432 (3)
N3—C18	1.153 (3)	C16—C18	1.432 (3)
N4—C20	1.143 (3)	C19—C20	1.432 (3)
N5—C21	1.147 (3)	C19—C21	1.446 (3)
C1—C2	1.392 (3)	C2—H2	0.950
C1—C6	1.393 (3)	C3—H3	0.950
C2—C3	1.388 (3)	C4—H4	0.950
C3—C4	1.388 (3)	C5—H5	0.950
C4—C5	1.392 (3)	C8—H8	0.950
C5—C6	1.389 (3)	C9—H9	0.950
C7—C8	1.394 (3)	C10—H10	0.950
C7—C12	1.395 (3)	C11—H11	0.950
C8—C9	1.389 (3)	C15—H15A	0.980
C9—C10	1.391 (3)	C15—H15B	0.980
C10—C11	1.396 (3)	C15—H15C	0.980
			
C6—S1—C7	97.49 (9)	C13—C16—C18	119.09 (16)
C1—N1—C12	113.32 (13)	C17—C16—C18	113.70 (15)
C1—N1—C13	120.28 (14)	N2—C17—C16	174.02 (18)
C12—N1—C13	123.30 (14)	N3—C18—C16	178.0 (2)
N1—C1—C2	121.70 (15)	C14—C19—C20	122.01 (18)
N1—C1—C6	116.87 (15)	C14—C19—C21	120.99 (18)
C2—C1—C6	121.43 (15)	C20—C19—C21	116.93 (17)
C1—C2—C3	118.95 (17)	N4—C20—C19	179.1 (3)
C2—C3—C4	119.83 (17)	N5—C21—C19	178.0 (2)
C3—C4—C5	120.88 (17)	C1—C2—H2	120.520
C4—C5—C6	119.61 (17)	C3—C2—H2	120.526
S1—C6—C1	119.39 (13)	C2—C3—H3	120.088
S1—C6—C5	121.62 (14)	C4—C3—H3	120.079
C1—C6—C5	118.99 (16)	C3—C4—H4	119.564
S1—C7—C8	121.29 (14)	C5—C4—H4	119.554
S1—C7—C12	119.38 (13)	C4—C5—H5	120.189
C8—C7—C12	119.32 (16)	C6—C5—H5	120.199
C7—C8—C9	119.11 (17)	C7—C8—H8	120.448
C8—C9—C10	121.28 (17)	C9—C8—H8	120.446
C9—C10—C11	119.94 (18)	C8—C9—H9	119.360
C10—C11—C12	118.51 (17)	C10—C9—H9	119.363
N1—C12—C7	116.93 (15)	C9—C10—H10	120.029
N1—C12—C11	121.16 (15)	C11—C10—H10	120.035
C7—C12—C11	121.71 (16)	C10—C11—H11	120.744
N1—C13—C14	114.81 (14)	C12—C11—H11	120.746
N1—C13—C16	127.51 (16)	C14—C15—H15A	109.470
C14—C13—C16	117.63 (14)	C14—C15—H15B	109.468
C13—C14—C15	117.89 (15)	C14—C15—H15C	109.466
C13—C14—C19	119.08 (16)	H15A—C15—H15B	109.474
C15—C14—C19	123.01 (17)	H15A—C15—H15C	109.475
C13—C16—C17	127.17 (15)	H15B—C15—H15C	109.474

### Hydrogen-bond geometry (Å, º)

**Table:**

*D*—H···*A*	*D*—H	H···*A*	*D*···*A*	*D*—H···*A*
C15—H15*B*···N4^i^	0.98	2.65	3.186 (3)	114
C2—H2···N5^ii^	0.95	2.59	3.352 (3)	137
C10—H10···N2^iii^	0.95	2.70	3.413 (3)	133
C8—H8···N2^iv^	0.95	2.62	3.480 (3)	151

Symmetry codes: (i) *x*, *y*+1, *z*; (ii) −*x*, *y*−1/2, −*z*+1; (iii) −*x*+1, *y*−1/2, −*z*; (iv) −*x*+1, *y*+1/2, −*z*.
